# Conscious Sedation and Analgesia in Colonoscopy: Ketamine/Propofol Combination has Superior Patient Satisfaction Versus Fentanyl/Propofol

**DOI:** 10.5812/aapm.9653

**Published:** 2013-07-01

**Authors:** Mohammadreza Khajavi, Azra Emami, Farhad Etezadi, Saeid Safari, Alireza Sharifi, Reza Shariat Moharari

**Affiliations:** 1 Department of Anesthesiology, Sina Hospital, Tehran University of Medical Sciences, Tehran, Iran; 2 Department of Anesthesiology, Rasoul Akram Medical center, Iran University of Medical Sciences (IUMS), Tehran, Iran; 3 Department of Gastroenterology, Sina Hospital, Tehran University of Medical Sciences, Tehran, Iran

**Keywords:** Colonoscopy, Ketamine, Propofol, Fentanyl, Patient Satisfaction

## Abstract

**Background:**

Colonoscopy is performed without preparing sedation in many countries. However, according to the current literature patients are more satisfied when appropriate sedation is prepared for them.

**Objectives:**

We hypothesize that propofol-ketamine may prepare more patient satisfaction compared to propofol-fentanyl combination.

**Patients and Methods:**

Sixty adult patients older than 18 with ASA physical status of I, II or III were enrolled in the present study after providing the informed consent. They were prospectively randomized into two equal groups: 1- Group PF: was scheduled to receive IV bolus dose of fentanyl 1µg/kg and propofol 0.5mg/kg. 2- Group PK: was scheduled to receive IV bolus dose of ketamine 0.5mg/kg and propofol 0.5mg/kg. As a primary goal, patient’s satisfaction was assessed by the use a Likert five-item scoring system in the recovery. Comparisons of hemodynamic parameters (mean heart rate, mean systolic blood pressure, mean diastolic blood pressure), mean Spo2 values during the procedure and side effects such as nausea, vomiting, and psychological reactions during the recovery period were our secondary goals. Level of sedation during the colonoscopy was assessed with the Observer’s Assessment of Alertness/Sedation score (OAA/S).

**Results:**

Mean satisfaction scores in the group PK were significantly higher than the group PF (P = 0.005) while the level of sedation during the procedure was similar (P = 0.17). Hemodynamic parameters and SpO2 values were not significantly different (P > 0.05). Incidence of nausea and vomiting was the same in both groups.

**Conclusions:**

IV bolus injection of propofol-ketamine can lead to more patients’ satisfaction than the other protocols during colonoscopy.

## 1. Background

Colonoscopy is one of the most commonly performed outpatient procedures throughout the world as a screening, diagnostic, and therapeutic tool. The pain and anxiety which are frequently associated with colonoscopy, may lead to either patient refusal or elevated medication administration (1). Routine colonoscopy can be performed without preparing sedation in many countries (2). The reason may be fear of probable cardio-respiratory complications or a high cost of anesthesia facilities. Since either moderate or light level of anesthesia can provide adequate pain control and hypnosis for most patients, it is recommended to avoid a deep level of sedation in these patients (3, 4). It must be noted that propofol combined with narcotic drugs are used widely for sedation during colonoscopy. Although, this combination may increase patient comfort, but because of the synergistic depressive effect of this combination on cardio-respiratory system, occasionally cardio-respiratory adverse events may take place (5). Ketamine produces dose-related unconsciousness and analgesia with minimal effect on the central respiratory drive while stable hemodynamics are maintained (6, 7).

## 2. Objectives

Since no study has evaluated the role of bolus injection of ketamine as an analgesic component of anesthesia in comparison with the other analgesics (opioids) in colonoscopy procedures and because of the increasing importance of patient’s satisfaction and preparation of analgesia during invasive medical interventions we designed this study to compare the effects of, ketamine-propofol versus fentanyl-propofol for achieving a more acceptable satisfaction of the patients during colonoscopy procedures.

## 3. Patients and Methods

This was a double-blind, prospective, randomized controlled trial conducted in the Endoscopy Center in the Sina Hospital of the Tehran University of Medical Sciences between March 2010 and May 2011. The university ethics board approved this study and all participants provided an informed consent. Sixty ASA physical statuses I, II or III patients who were older than 18 years were included in this study. Randomization of patients was performed by the use of a sealed envelope technique. Patients who had a recent history of colonoscopy, a previous colonic resection, severe heart failure (ejection fraction < 30%) and known history of hypersensitivity to midazolam, propofol, ketamine or fentanyl were excluded from the study. In addition, any need for further anesthetic drug administration other than the study protocol was another exclusion criterion of this study. Sedation for colonoscopy was administered by an attending anesthesiologist (cooperated with resident of anesthesiology) who was blinded from drug allocation. The colonoscopies were performed by a gastroenterologist who was blinded from the type of drugs used for sedation. The study was performed. In the endoscopy room, for all patients after establishment of intravenous access, standard monitoring (noninvasive blood pressure, electrocardiography and pulse oximetry) was performed. Use of standard monitoring continued in the recovery unit, until the patients were discharged. Oxygen (6 l/min) administration commenced via facemasks for all patients and, midazolam 0.03mg/kg IV as a premedication was injected to all of the patients. By the means of a double-blind randomized construct, patients were scheduled to receive either IV bolus dose of fentanyl (Fentanyl 0.5 mg/10ml, Aburaihan Co. Iran) (1µg/kg) and propofol (Propofol 1% MCT/LCT Fresenius, manufactured by Fresenius Kabi Austria.) (0.5mg/kg) in group PF or ketamine (Ketamine Hydrochloride 500mg/10 ml, Rotexmedica, TRITTAU, Germany) (0.5mg/kg) and (propofol 0.5mg/kg) in the group PK. The serious adverse events during the study period were defined as: 1- >30% change in baseline systolic blood pressure (SBP); 2- > 30% change in diastolic blood pressure (DBP); 3- HR < 50/min; 4- apnea > 30 sec; 5- SpO_2 _< 85%. Treatment of the aforementioned adverse events was at the discretion of the anesthesiologist caring for the patient. The level of sedation (an objective variable) during the procedure was assessed with an Observer’s Assessment of Alertness/Sedation (OAA/S) scores (1 = fully sedated, 5 = not sedated) every five minutes after commencement of sedation (four times during the procedure) (8). After the end of the procedure, in the recovery ward and when the patients were alert enough to express their attitude regarding the intra-procedural events, they were asked to score their level of satisfaction during the procedure in terms of recalling any painful or other undesirable intra-procedural events. Patient’s satisfaction level was assessed with a Likert five-item scoring system (1 = Not at all satisfied, 2 = slightly satisfied, 3 = somewhat satisfied, 4 = very satisfied, and 5 = extremely satisfied) (9). All OAA/Sand Likert scores were obtained by one investigator (who was blinded to the drug allocation) to reduce inter observer variability. Hemodynamic parameters (heart rate, systolic blood pressure, diastolic blood pressure) and SpO_2_ values were recorded every five minutes (three times during the procedure). Probable side effects (nausea and vomiting, psychological reactions) were noted during recovery period as well. We used the Aldrete’s scoring system for the discharge of patients from recovery. Achievement of at least 8 out of 10 scores was the criteria for discharge in this study (10). The primary aim of this study was to compare the means of patients’ satisfaction scores after the end of procedure (in the recovery period) between the patients of the two groups. Comparison of hemodynamic parameters (mean heart rate, mean systolic blood pressure, and mean diastolic blood pressure) and mean SPO_2_ values throughout the procedure and also some probable side effects such as nausea, vomiting and psychological reactions during the recovery period between groups was our secondary objective.

### 3.1. Statistical Analysis

All quantitative data were expressed as means ± standard deviation (SD) and compared using student T test. For comparison of categorical data, K2 test was used. All qualitative data were expressed as numbers (%) and compared with the Fisher's exact probability test. For comparison of the sedation score data, hemodynamic parameters and SpO_2_ values, the repeated measurement analysis was used. A sample size of thirty patients in each group was calculated to have at least an 80% power to detect the expected differences between the two groups with respect to the primary goal. Finding a difference of at least two out of five in the mean satisfaction scores (40% change) between the two groups was regarded as a clinically significant difference. A P value less than 0.05 was considered as statistically significant.

## 4. Results

**Figure 1.**
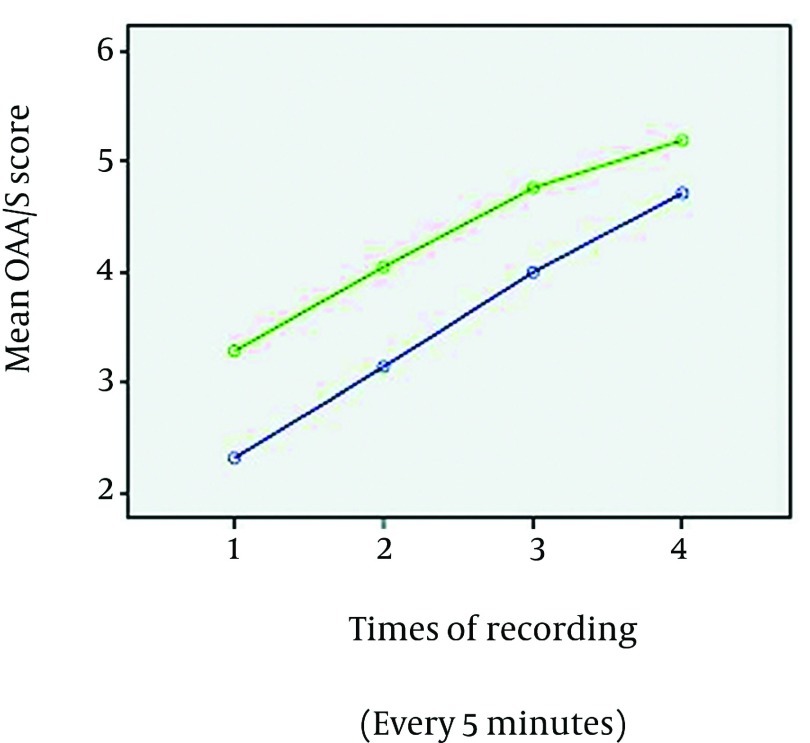
Comparison of Changes in the Mean Sedation Scores in the Two Groups, Throughout the Colonoscopy Blue line = group PK, Green line= group PF, OAA/S = Observer’s Assessment of Alertness/Sedation.

**Figure 2.**
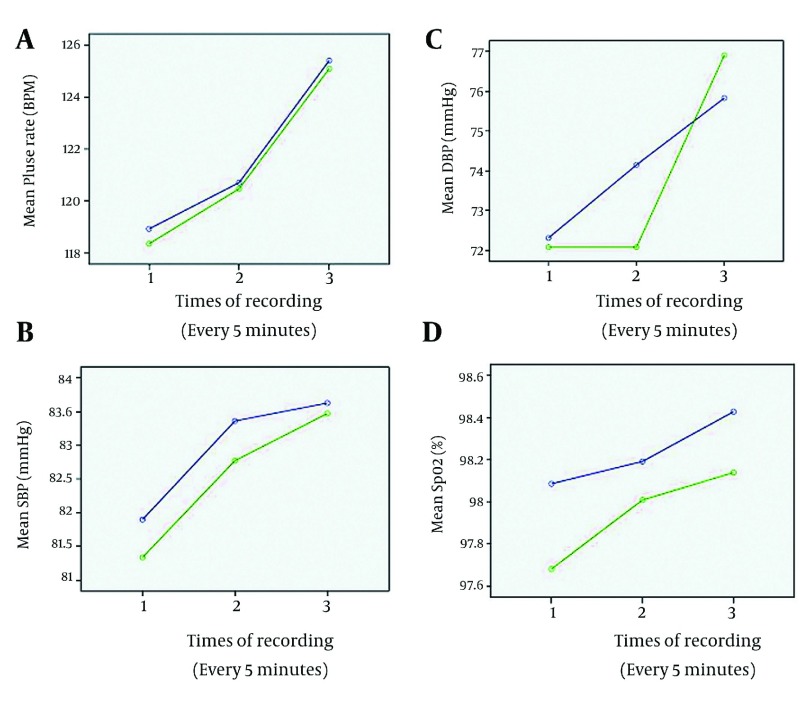
Comparison of changes in: Mean Pulse Rate (A), Mean Systolic Blood Pressure (B), Mean Diastolic Blood Pressure (C), and mean SPO2 (D) throughout the colonoscopy. Blue line = group PK, Green line= group PF

Sixty patients were enrolled in the study, 30 in each group. No predefined serious adverse events were observed in the patients of both groups. No patient during the procedure was excluded from the study because of extra-protocol sedative administration. No significant demographic differences were identified between the two groups (Table 1).The mean duration of colonoscopy in the group PK was 23.3 ± 7.7 minutes and it was 21.8 ± 9.7 minutes in the group PF. The mean duration of recovery in the group PK was 50.6 ± 6.2 minutes and it was 45.3 ± 8.4 minutes in the group PF. Although, the patients in the group PK had lower mean sedation scores (more sedated) during the procedure but this was not statistically significant between the two groups (P = 0.17) (Figure 1). The mean of Likert satisfaction scores of the patients in the group PF was1.8 ± 0.4 (mode = 2), while it was 3.9 ± 0.5(mode = 4) in the group PK. The mean Likert satisfaction scores of the patients were significantly higher in the PK group (P = 0.005). The trend of hemodynamic variables, (SBP, DBP, and HR) during the procedure was similar between the two groups (P > 0.05). In addition, the trend of SPO_2_ changes remained similar throughout the colonoscopy in the two groups (P > 0.05) (Figure 2). The incidence of complications such as nausea and vomiting were similar in the two groups (12.5%). During the recovery period, three patients had psychological emergence reactions in the group PK (7.5%), but, this was self-limited and didn’t need any medication.

**Table 1.** Characteristics of Included Patients and the Procedures

Variables	Group PF ^a^	Group PK ^a^
Age, y, Mean ± SD	51.6 ± 21	55.9 ± 15
Sex (Male/Female), Mean ± SD	18/12	16/14
Weight, kg	56 ± 14	59 ± 17
Height, cm	155 ± 5	157 ± 8
ASA ^a^ physical status		
I	12	14
II	12	9
III	6	7
Duration of Procedure, min, Mean ± SD	21.8 ± 9.7	23.3 ± 7.7
Duration of Recovery time, min, Mean ± SD	45.3 ± 8.4	50.6 ± 6.2

^a^Abbreviations: ASA, American society of anesthesiologists; PF, propofol-fentanyl; PK, propofol-ketamine

## 5. Discussion

This is the first study, which evaluates bolus IV injection of a ketamine-propofol combination in comparison with fentanyl-propofol combination in the colonoscopy procedure.

The principal result of this investigation is that patients in the PK group have been more satisfied (in terms of recalling any undesirable experiences such as pain or discomfort) than the other group. The sedation scores during the procedure were comparable between the two groups; in addition, the trend of hemodynamic and respiratory variables was similar in the two groups. Sedation for colonoscopy should provide an optimal hypnosis and analgesia with a lower probability of hemodynamic and respiratory complications; thus, drug selection is a crucial determinant of these outcomes. According to the study by Fanti et al, the most common complications in gastrointestinal endoscopy are not related to the procedure, but are related to sedation; they include cardio-respiratory adverse events such as hypoxemia, hypoventilation, apnea, dysrhythmias, hypotension and vasovagal episodes (11). Fortunately, in this study no adverse events were seen during the colonoscopies of either group. The achieved sedation in the two groups of study was at a moderate level (Figure 1), thus, it is not surprising that no cardiorespiratory complication was observed. Since several researchers have found propofol, as a hypnotic drug, to be superior to traditional sedative regimens (because of rapid recovery), the use of propofol for endoscopic sedation has been increased significantly during the past 10 years (11-16). Propofol in combination with midazolam can be titrated to achieve a moderate level of anesthesia in colonoscopy, but it is important to note that this combination lacks analgesic properties and may result in the sensation of more pain and consequently a low level of patient satisfaction (17). The mean of procedure duration was similar in patients of both groups (21 versus 23 minutes) in this study, similar to Sipe and Paspatis’studies (15, 16). The frequency of nausea in patients who were sedated with propofol plus narcotics in the Kostash et al. study was 26 %, (range 16-40%) while it was much less (12.5%) in our study (18). The combination of ketamine and propofol for performing procedural sedation theoretically may be advantageous as using lower doses of each agent may result in a reduction of the adverse drug effects while maintaining an acceptable condition for colonoscopy. It is noteworthy to mention that a low plasma level of ketamine can inhibit nociceptive central sensitization and has a preemptive analgesic effect (19, 20). The use of propofol– ketamine infusion for procedural sedation and analgesia outside the operating room environment especially in the emergency department and pediatric patients has become popular (21, 22). However, researchers have found insufficient data to recommend the use of the aforementioned combination. Additionally, Slavik et al. did not support the use of a bolus dose of propofol-ketamine for procedural sedation and analgesia (23). On the contrary, the results of this study revealed that injecting a bolus dose of propofol-ketamine is not only an acceptable sedative option, but also it may be superior to the other commonly used combination (propofol-fentanyl) for sedation of patients during the colonoscopy procedures. On the other hand, ketamine may produce undesirable psycho- mimetic reactions, known as "emergence reactions", which may occur during awakening from anesthesia. Factors that affect the incidence of emergence reactions are age, dose, gender, psychological susceptibility, and concurrent drugs (7). Additionally, it was suggested that psycho- mimetic reactions may occur predominantly in the case of a large dose injection of ketamine (24). The incidence of this reaction in our study was low (7.5%). The reason may be injection of midazolam to all patients. In addition, the combination of ketamine with propofol may be the other factor, which contributed to the low incidence of psycho- mimetic reactions of ketamine in this study. This study may be subjected to one limitation: the study duration was short because of some logistical reasons; it might be better to follow patients until 24 h to discover probable adverse events which may occur even after discharge. Therefore, we suggest that more research is needed to elucidate the role of this combination for sedation and analgesia in colonoscopy procedure. For preparing sedation in colonoscopy procedure, use of a bolus IV dose of ketamine-propofol is more acceptable than the fentanyl- propofol combination.
